# A quality improvement initiative to improve transport team mobilization for emergent neonatal transfers

**DOI:** 10.3389/fped.2025.1623047

**Published:** 2025-07-23

**Authors:** Arpit Sohane, Arshdeep Kaur, Pradeep Suryawanshi, Arjun Verma, Suprabha Patnaik

**Affiliations:** ^1^ Department of Neonatology, Bharati Vidyapeeth Deemed University Medical College and Hospital, Pune, India; ^2^ Department of Neonatology, Mahatma Gandhi Medical College, Mahatma Gandhi University of Medical Sciences and Technology, Jaipur, India

**Keywords:** neonate, emergent transfer, mobilization time, quality improvement, LMIC

## Abstract

**Introduction:**

A considerable number of neonatal deaths can be attributed to delays in accessing appropriate medical care or the absence of reliable systems for transferring newborns to advanced care centers. Infants requiring urgent transfer to specialized neonatal units are particularly vulnerable, often due to the limited capabilities of peripheral birthing facilities, the absence of structured neonatal transport networks, and the reliance on unsafe or uncoordinated transfer methods. Approximately 25% of neonatal transfers are time-sensitive and require specialized neonatal teams to respond promptly. Any delay in the mobilization of the retrieval team can adversely affect the delivery of this essential time-sensitive care.

**Methods:**

This single-center quality improvement (QI) study was conducted at a tertiary care center with a Level 3B NICU. We aimed to reduce our MT to ≤10 min in >70% of emergent neonatal transfers within 3 months. Multiple change ideas and Plan-Do-Study-Act (PDSA) cycles (designation of transport nurse, education regarding timely mobilization, keeping prechecked retrieval kit, keeping all equipment together) were carried out.

**Results:**

MT was reduced to ≤10 min in 70.6% of transfers, and MT was under 15 min in 80.6% of emergent neonatal transfers, achieving the benchmark of 15 min given for “launch time” by the Australian New Zealand Neonatal Retrieval Network (ANZNRN) 2022 data dictionary.

**Conclusion:**

Effectively formulated and executed QI strategies can accelerate the benchmark time-related quality indicators for urgent neonatal transport teams during critical neonatal transfers. Our study encourages other healthcare setups to improve MT through simple yet effective measures.

## Introduction

The Sustainable Development Goals (SDGs) aim for all countries to achieve an under-5 mortality rate (U5MR) of ≤25 deaths per 1,000 live births by 2030 (SDG target 3.2.1) and a neonatal mortality rate (NMR) of ≤12 deaths per 1,000 live births by 2030 (1). Of the 140 million live births globally, approximately 2.5 (2.2–2.7) million neonates die. Despite two decades of global initiatives, such as the Millennium Development Goals and the SDGs, the neonatal period continues to represent the highest risk of mortality in early childhood. Global neonatal mortality accounts for approximately 50% of the U5MR, with the majority occurring in low-and middle-income countries (LMIC). As of 2019, nearly 80% of global neonatal deaths occurred in sub-Saharan Africa and Southern Asia, with two-thirds taking place on the first day of life and almost three-quarters within the first week (2).

A significant proportion of neonatal deaths result from delays in timely engagement with the healthcare system or the absence of efficient transfer mechanisms to higher-level care facilities. Critically ill newborns face an elevated risk of adverse outcomes, often compounded by the unavailability of dedicated transport services and the limited resources at peripheral healthcare centers. Ensuring prompt, appropriate, and timely referral of these newborns has the potential to substantially reduce the high mortality rates observed on the day of birth (3). Improving neonatal survival requires ensuring that all critically ill newborns have immediate access to appropriate healthcare facilities. In LMICs, a significant number of preterm births occur in peripheral centers that are often ill-equipped to manage neonatal complications. The deployment of specialized neonatal transport teams can bridge this gap by delivering timely, high-quality critical care during transfers, thereby reducing both neonatal mortality and morbidity in the community.

Approximately 25% of urgent neonatal transfers, which pose a significant risk to life or organ function, are classified as time-sensitive (4). Such transfers necessitate the prompt response of specialized neonatal teams. Any delays in the departure of the retrieval team from their base can adversely affect the delivery of this essential time-sensitive care. The definitions of activation time for the transfer team vary according to the organization. The Australian New Zealand Neonatal Retrieval Network (ANZNRN) 2022 data dictionary refers to this as “launch time,” while the Ground and Air Medical Quality Transport (GAMUT) 2015 database refers to this as “mobilization time” (MT) (5). This serves as a crucial time-dependent quality metric for establishing benchmarks. MT is a standardized measure used to monitor the quality of care the transport team provides. It is defined as time in minutes from the start of the referral phone call to the time the transport team is en route to the referral facility. MT is influenced by various factors such as the availability of ambulances, simultaneous retrievals, the location of the NICU and departure area, and team communication. The GAMUT database suggests a benchmark of 25 min for MT (5). Analysis of our baseline data revealed that the MT for emergent neonatal transfers frequently exceeded 30 min, contributing significantly to delays in critical care delivery. Recognizing the importance of timely mobilization, we initiated this quality improvement (QI) project to reduce our MT and enhance the efficiency of neonatal transport.

## Methods

### Study design: single-center QI study

#### Study settings

The neonatology unit where this study was conducted is attached to a medical college and comprises 19 intensive care level III beds, 21 step-down level II beds, and 20 beds in the Level I mother–baby area. The unit caters to approximately 1,500 admissions annually, out of which 10%–15% are retrieved as extramural babies by the transport team, which covers a radius of approximately 200 km. The longest journeys extend approximately 250 km and can require between 6 and 8 h for completion. Each retrieval team consists of doctors (senior resident or fellow in neonatology, along with a junior resident in pediatrics), a nurse (neonatal trained), and support staff (multipurpose worker and ambulance driver).

#### Aim

To evaluate the feasibility of various target mobilization times, we conducted several trial runs. Based on these assessments, we determined that a 10 min MT was achievable for the transport team to depart from our facility to the referring hospital. Consequently, our QI goal was to reduce the MT to ≤10 min in >70% of emergent neonatal transfers within a 3-month period.

#### Team formation and process mapping

**Figure 1:**
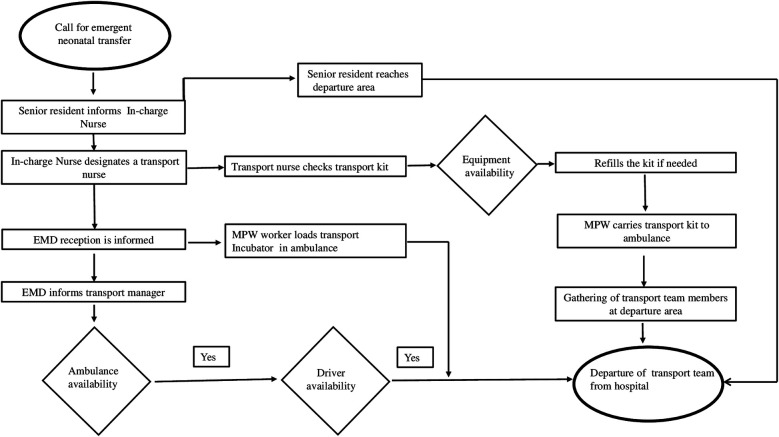
Process mapping.

**Figure 2:**
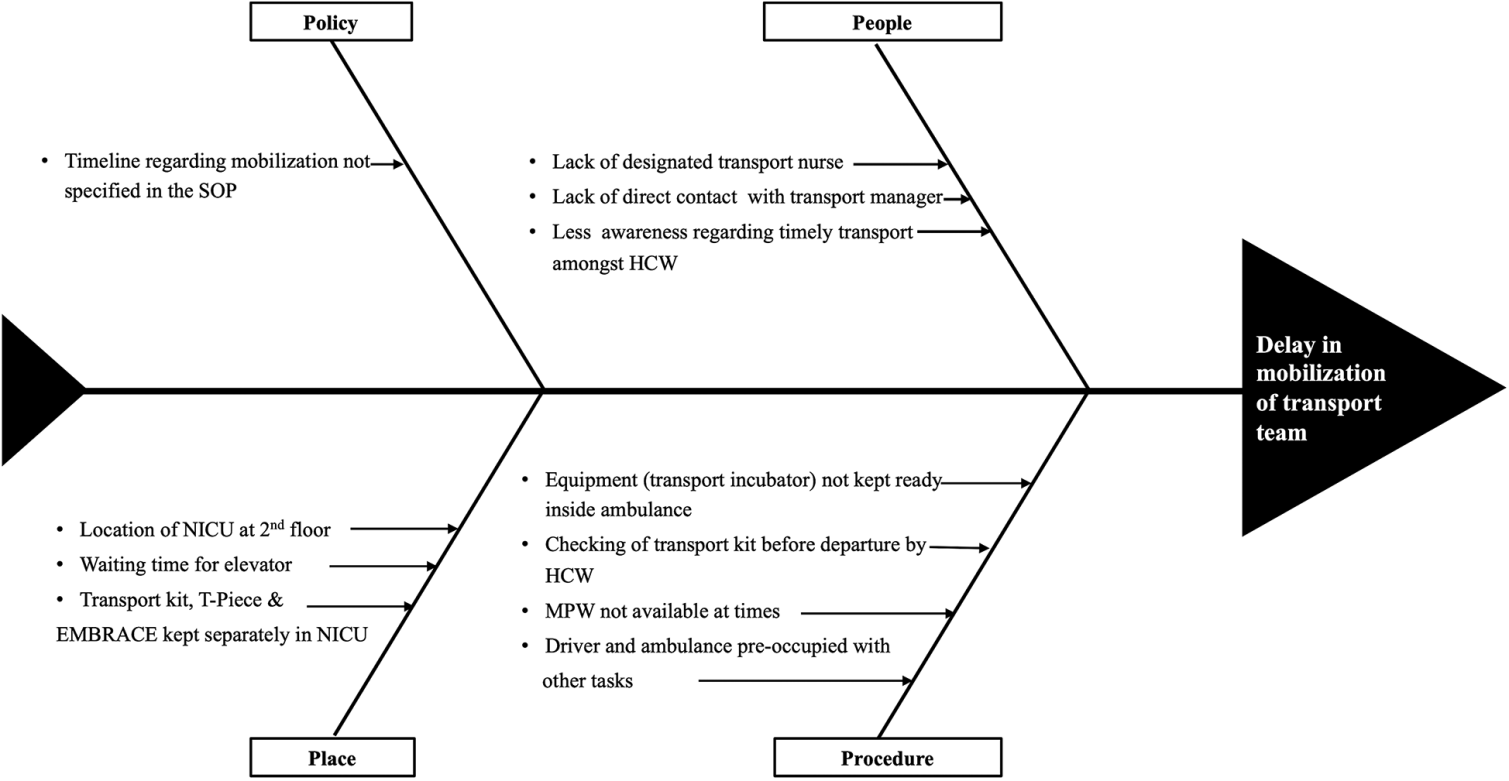
Fish bone analysis.

A multidisciplinary team comprised of neonatology, nursing, emergency medicine, transport, and housekeeping departments. Interdepartmental meetings and group discussions were held among team members and stakeholders. Process mapping was done to gain insight into existing systems and processes (Figure 1). This helped us to analyze probable causes of delay in mobilization and modifiable factors (Figure 2).

#### Strategy

We followed the WHO's Point of Care Quality Improvement (POCQI) model for this QI project (6). Multiple Plan-Do-Study-Act (PDSA) cycles as part of the QI initiative were introduced during the study. Interdepartmental meetings and group discussions were held at the beginning of each PDSA cycle. The sustainability of the intervention was ensured in the long run through repeated reinforcement and educational sessions.

### PDSA Cycle 1

#### Designation of a transport nurse at the beginning of the shift

Prior to the initiation of this quality improvement project, the transport nurse was designated by the nursing in-charge on a rotational basis only after a transfer call was received. The selected nurse would then need to hand over the care of her assigned patient—often a critically ill neonate receiving life-supportive therapy—before proceeding with the transfer. This process introduced significant delays in mobilization.

Following the implementation of the QI initiative, a dedicated “transport nurse” was pre-assigned at the start of each duty shift and was intentionally allocated a relatively stable patient to facilitate immediate availability for transport. This change was piloted over 30 shifts (3 shifts per day for 10 days) and was found to be feasible, with 100% compliance during the trial period. The intervention effectively eliminated the time required for last-minute nurse assignment and patient handovers, thereby contributing to a reduction in MT. This strategy was subsequently adopted as a standard practice, with the name of the designated transport nurse visibly displayed on the unit board.

### PDSA Cycle 2

#### Education

To reinforce the importance of timely neonatal mobilization, regular education and training sessions were conducted through 30 min lectures utilizing audiovisual aids. These sessions were held weekly over a 3-week period and were led by a senior resident in neonatology. Attendance included fixed nursing staff, newly rotated nursing personnel, and junior residents undergoing their NICU posting as part of their postgraduate training in pediatrics.

Multipurpose workers were also oriented regarding their specific roles in minimizing MT. These sessions fostered greater awareness and a sense of urgency among all team members, encouraging prompt preparation and departure for neonatal transfers. The sessions were attended by 80% of the designated staff, as these were conducted during the overlap of the morning and evening nursing shifts.

### PDSA Cycle 3

#### Prechecked retrieval kit

The neonatal retrieval kit is equipped with all essential items required to stabilize and support the newborn during transport, including supplies for thermal regulation (e.g., warm linen and woolen head cap), airway and respiratory management (e.g., shoulder roll, AMBU bag with oxygen reservoir, face masks of various sizes, laryngoscope with blades, and endotracheal tubes), and non-invasive ventilation interfaces. It also contains medications commonly used during transport, such as intravenous dextrose, normal saline, and critical emergency drugs, including inotropes.

Prior to the QI initiative, this kit was usually inspected only after a retrieval call was received, contributing to delays in mobilization. As part of the QI interventions, the designated transport nurse began checking the retrieval kit at the start of each shift and replenishing supplies as needed. This practice was implemented over 30 consecutive shifts (3 shifts per day for 10 days) and was found to be feasible. The intervention led to a reduction in MT by approximately 3–4 min and was subsequently incorporated into the standard operating protocol.

### PDSA Cycle 4

#### Placement of all equipment necessary for retrieval together

Previously, key transport equipment—including the retrieval kit, T-piece resuscitator, and the thermal transport mattress (EMBRACE™)—were stored in separate locations within the NICU, based on feasibility and available space. As a result, following a retrieval call, the transport nurse needed to collect these items from different areas before mobilization, causing an additional delay of approximately 2–3 min for each transfer.

As part of the QI initiative, all essential transport equipment was relocated and stored together in a designated, easily accessible area. This change was implemented over a 2-week period and proved to be both feasible and effective. The practice significantly reduced MT and was subsequently adopted as part of the standard workflow.

#### Ongoing education

Weekly sessions will be held to educate new rotating staff nurses and junior residents about the retrieval process and MT, as described in PDSA Cycle 1, to maintain the project's continuity.

#### Data collection

Baseline data were collected using the “retrieval register” already existing in the system**.** A group on WhatsApp software for mobile devices was formed for this initiative to keep all stakeholders in the loop. Details of each neonatal transfer, such as name and designation of transport team members, location of the destination hospital, time of the call received, time when the transfer team left the NICU, and time when the transfer team departed the hospital, were shared on the group. Patient identifiers were not shared with the group. Any problems, shortcomings, and reason/s for delay (if any) in departure were also shared. The data were initially curated and reviewed on a weekly basis and then transitioned to monthly evaluations. Any identified delays were addressed within 24 h. Additionally, weekly meetings were held every Friday within the department to discuss the issues, identify root causes, and implement corrective actions.

#### Outcome indicator

The outcome was measured as the percentage of emergent neonatal transfers with MT within 10 min, calculated by dividing the total number of neonatal transfers with MT within 10 min by the total number of confirmed calls for emergent neonatal transport, multiplying by 100.

## Results

**Figure 3:**
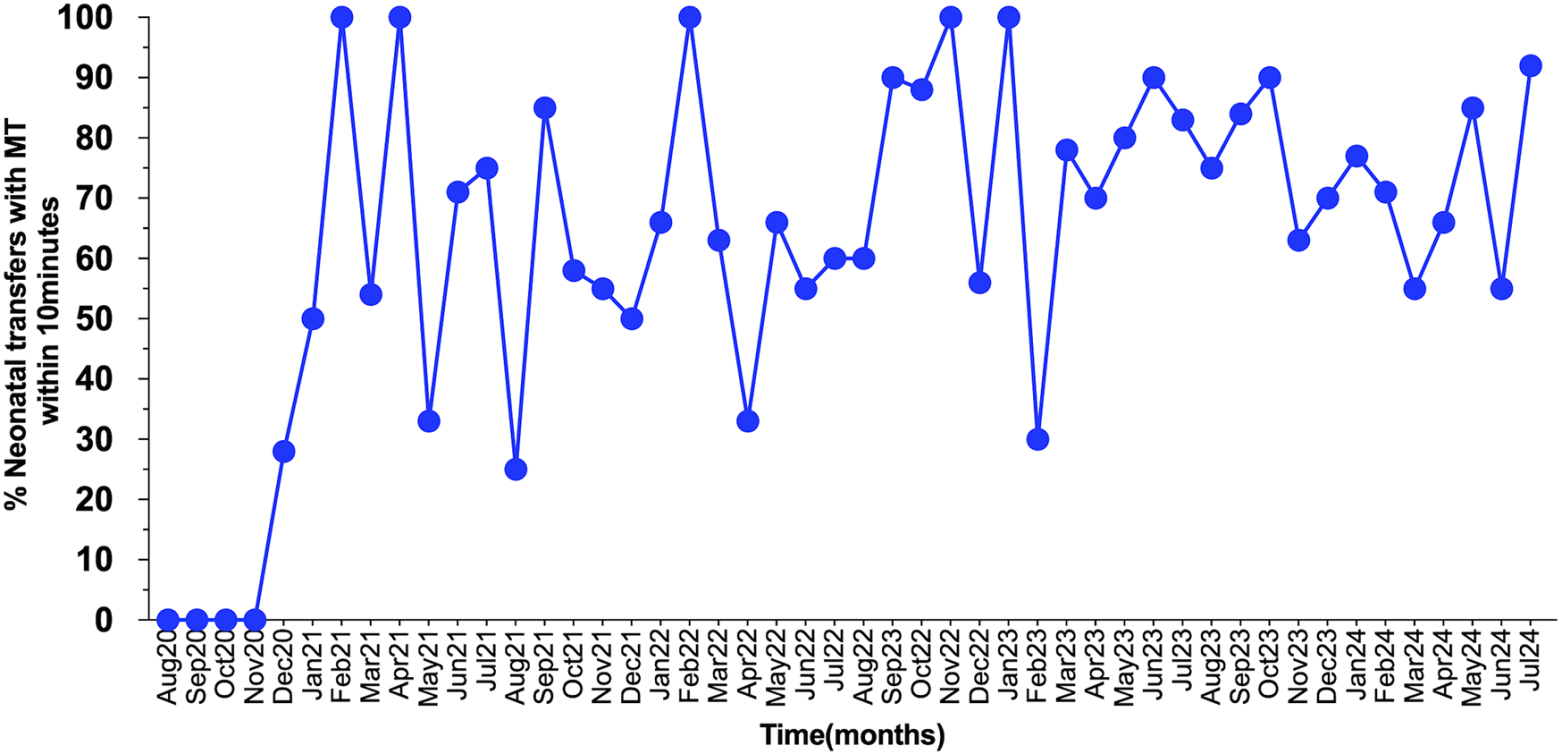
The statistical process control chart demonstrating the percentage of mobilizations within 10 min during study period.

**Figure 4:**
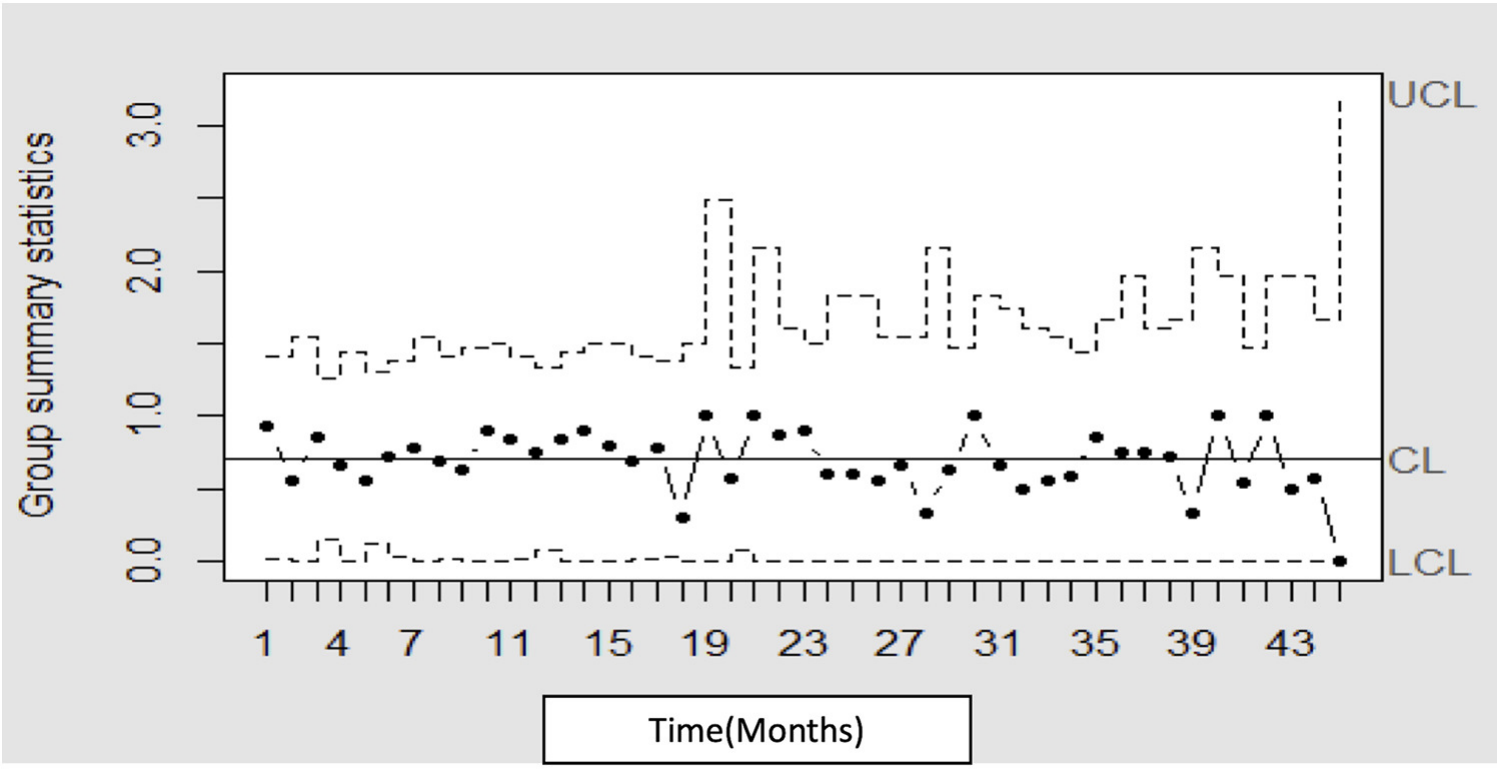
The statistical process U-chart demonstrating that the quality indicator is well under statistical control limits.

**Figure 5:**
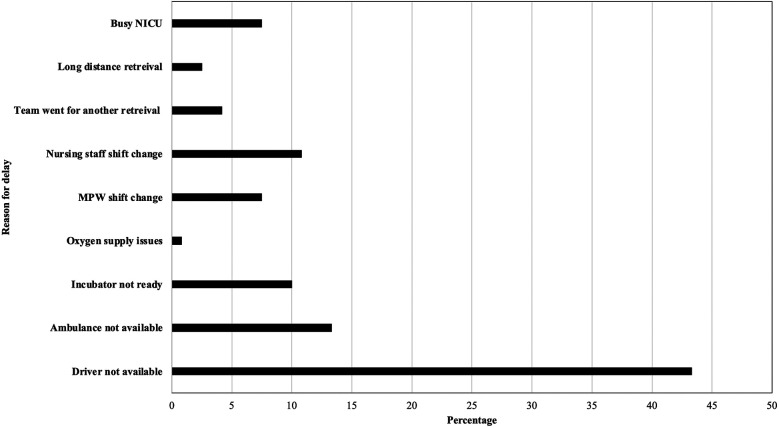
Pareto analysis of factors contributing to delay in mobilization.

Between November 2020 and July 2024, a total of 409 neonates were transported. As a result of the QI initiatives, MT was reduced to 10 min or less in 70.6% of transfers and to under 15min in 80.6% of emergent neonatal transfers (Figure 3). This achievement met the established benchmark of 15 min for “launch time” (same as mobilization time by definition) by the Australian New Zealand Neonatal Retrieval Network (ANZNRN) 2022 data dictionary (7). A U-chart derived from the data using R V.4.3.1 (R Core Team, 2023) demonstrated that the quality indicator is well under statistical control and all data points fall within control limits (Figure 4). We evaluated reasons for delay in MT using Pareto analysis which helped us design interventions to decrease our MT in future transfers (Figure 5). The majority of the delays were identified due to non-modifiable factors at our departmental end such as non-availability of ambulance and/or driver at the time of emergent transfer call.

### Post-PDSA period

This project is now in a sustainable phase, and MT has become a QI indicator in our unit.

## Discussion

Numerous neonatal transport networks worldwide have established quality metrics focused on time for benchmarking purposes. These metrics exist individually for safety and effectiveness during transport, as well as for timely, equitable, and efficient transport, and metrics focusing on patient-centered care. Some of these metrics are enumerated in Table 1. The most important quality domain among all is timeliness; however, transport time as a quality metric has inconsistencies in its definition, as different publications, organizations, and reports use different nomenclature and definitions (8).

**Table 1:** Various quality metrics for neonatal transport.

Quality domain	Ground and air medical quality in transport
*Safety*	• Dislodgement of therapeutic devices • Crew/Patient injury • Medication errors • Medical equipment failure • Serious reportable events
*Effective*	• Hypothermia (temperature <36.5℃ axillary at destination site) • First attempt at successful tracheal tube placement • Rapid sequence intubation • CPR during transport • Hypoxia during transport (saturation below 90%)
*Patient-centered*	• Reliable pain assessments
*Timely*	• Mobilization time
*Efficient*	• Use of standardized patient care handoff
*Equitable*	

There is considerable variability in establishing reference standards for benchmarking transport times. Transport time has been interchangeably defined as “response time” by some authorities (e.g., the Infant Transport Team of British Columbia defines “response” times of 15 min for transport requests of unstable patients by land and 1 h by air) (9).

There is a significant debate on what should be prioritized first: a shorter stabilization time or a shorter MT. Despite years of research, an optimal time for stabilization remains a mystery. Additionally, a shorter stabilization time was not found to be beneficial for neonates in terms of outcome, unlike the findings in the adult population. Due to their unique physiology, neonates require a longer stabilization time (median, 45 min) compared with pediatric or adult patients. Sicker neonates (those on inotropes or invasively ventilated) had a longer stabilization time than their relatively stable counterparts (10).

As previously noted, reduced stabilization time during transfers of adults and children does not produce comparable outcomes for neonates; therefore, QI initiatives aimed at facilitating faster mobilization would be more advantageous in urgent neonatal retrieval scenarios. The primary goal should be the expedited transfer of the sick neonate to a higher-level facility, rather than minimizing stabilization times, so that the neonate can benefit from advanced skills, infrastructure, modern equipment, and the latest technology. Acknowledging this fact, numerous QI studies have been conducted to date, encompassing various transport quality metrics, and in the study conducted by Rajapreyar et al. (11), average MT improved from 30.3 min to <25 min using the Prosci ADKAR model (awareness, desire, knowledge, ability, and reinforcement), five milestones to implement successful change. Their PDSA cycles included standardizing data collection and awareness building, engaging ambulance vendors, and implementing team-focused interventions. The non-availability of the retrieval team and a communication gap were the reasons for one-third of the delays prior to the implementation of their project. Arcinue et al. (4) achieved a target MT of <30 min in 82% of retrievals compared with 27%. Their PDSA cycles consisted of the provision of open critical bed space for incoming admission, faster patient room turnovers, improving communication skills, more transfer teams, and involving the accepting neonatologist with the referring and transport physicians on the initial call. In another QI study conducted by Kenningham et al. (12), MT decreased from 23 to 18 min after implementation of the QI project. Their PDSA cycles included a dedicated retrieval team and process mapping.

### Strength of the study

The present study has several strengths. The goal of achieving mobilization times of under 10 min was successfully met within a feasible period of 3 months and sustained for the past 3 years. The project is ongoing, with the exact timeline of MT as our set SMART aim. Very few QI studies have been conducted globally and in LMICs to improve MT for emergent neonatal transfers. Given that most neonatal transfers are conducted via road ambulances (road transfers) in LMICs, our study encourages other healthcare setups to improve their mobilization times through simple yet effective measures. The other strengths include regular focused group discussions with education sessions, which played a key role in maintaining team motivation.

### Lessons and limitations

The study had several limitations. Despite the implementation of the QI project, some non-modifiable factors still contributed to delays, such as the unavailability of a driver or ambulance and retrieval calls coinciding with shift changes of multipurpose workers or nursing staff. Another limitation was that our study focused solely on one quality metric—MT—and did not evaluate the impact of faster MT on stabilization time. Additionally, we did not correlate improved MT with clinical outcomes, as these outcomes are influenced by multiple variables, such as gestational age, birth weight, and cardiopulmonary status, at the time of retrieval.

## Conclusion and future implications

The present study highlights how small interventions can translate into significant change in results. Effectively formulated and executed quality improvement strategies can accelerate the benchmark time-related quality indicators for urgent neonatal transport teams during critical neonatal transfers. Analyzing the reasons for the delay was crucial and helped us deliver timely care. Future implications of this study involve evaluating multiple quality metrics in conjunction with mobilization time (MT). A reduction in MT, potentially reflecting improved clinical outcomes for patients, can be further assessed. Additionally, transport-related scores, such as the Transport Risk Index of Physiologic Stability (TRIPS) or the Mortality Index for Neonatal Transportation (MINT), could be integrated into future studies to provide a more comprehensive evaluation.

## Data Availability

The original contributions presented in the study are included in the article/Supplementary Material; further inquiries can be directed to the corresponding author.
